# Environmental Impacts of Glass- and Carbon-Fiber-Reinforced Polymer Bar-Reinforced Seawater and Sea Sand Concrete Beams Used in Marine Environments: An LCA Case Study

**DOI:** 10.3390/polym13010154

**Published:** 2021-01-02

**Authors:** Shaoce Dong, Chenggao Li, Guijun Xian

**Affiliations:** 1 Key Lab of Structures Dynamic Behavior and Control (Harbin Institute of Technology), Ministry of Education, Harbin 150090, China; 18846135354@163.com (S.D.); lichenggao02@126.com (C.L.); 2 Key Lab of Smart Prevention and Mitigation of Civil Engineering Disasters of the Ministry of Industry and Information Technology, Harbin Institute of Technology, Harbin 150090, China; 3 School of Civil Engineering, Harbin Institute of Technology, Harbin 150090, China

**Keywords:** life cycle assessment, environmental impacts, FRP bars, seawater and sea sand concrete

## Abstract

Application of glass- or carbon-fiber-reinforced polymer (GFRP/CFRP) bars makes the direct use of seawater and sea sand concrete (SWSSC) in construction feasible, which is of high interest in order to conserve the limited resources of fresh water and river sand. The present paper performed the life cycle assessment (LCA) of constructing three kinds of beams (GFRP/CFRP bar-reinforced SWSSC beams, and steel bar-reinforced common concrete (SRC) beam) in marine environments to show the environmental benefits of using FRP bar-reinforced SWSSC beams in marine environments. According to ISO 14040 and ISO 14044, stages including production, transportation, construction, use and end-of-life are within the LCA’s boundary. The ReCiPe method and eight main environmental impact categories were used to characterize the environmental impacts of those beams. LCA results indicate that one cubic meter SWSSC possesses much lower environmental impacts in terms of all eight categories compared with common concrete with the same volume when used in marine environments, with reduction rates from 26.3% to 48.6%. When the two transportation distances were set as 50 and 20 km and without considering the difference in service life, compared to SRC beam, GFRP-SWSSC beam performs better in six categories and CFRP-SWSSC beam performs better in four categories. When considering increased transportation distance and the higher durability performance, the advantageous categories for GFRP-SWSSC and CFRP-SWSSC beams increase to seven and six, respectively. The environmental impacts of all the three beams are mainly affected by the production stages.

## 1. Introduction

Concrete is one of the most commonly used construction materials because of its low cost and good durability [1]. However, application of concrete consumes great amount of fresh water, river sands, and crushed stones, bringing in severe resource depletion problems. In some areas of the world, resources of fresh water and river sands are bound to be used up due to heavy construction. For construction in marine areas, long transportation distance of river sands or crushed stones and production of desalinated seawater undoubtedly cause high costs and pollution. Therefore, replacing river sands and fresh water with sea sands and seawater is of great interest for the construction of structures under the corrosion of salt-laden air in coastal environments.

Seawater and sea sand concrete (SWSSC) has a large amount of chloride ions inside. Since heavy corrosion of traditional steel bars is expected to be occurred in SWSSC [2], SWSSC is generally strictly forbidden to be directly used for steel-reinforced concrete. Sea sands, washed with fresh water to reach a very low content of chloride ions (e.g., less than 0.03%), are permitted to be used for concrete in China [3]. Such treatment of sea sands or desalinating of seawater will definitely increase the costs and environmental burdens.

In view of the high contents of chloride ions in SWSSC, fiber-reinforced polymer (FRP) bars have been considered to be ideal to replace steel bars as reinforcements [4,5,6,7]. As an advanced structural material, FRP bars are made up of glass (GFRP), carbon (CFRP) or basalt fibers (BFRP) with epoxy or vinyl ester matrices. FRP bars possess many advantages, such as low density, high strength along fiber direction, and excellent corrosion resistance, etc. [8,9,10]. By now, construction codes for FRP bar-reinforced concrete structures have been published in many countries, such as China [11], USA [12], Japan [13] and Canada [14], etc.

There are many published research works about the workability, mechanical properties and durability of SWSSC. According to the critical review performed by Xiao et al. [15], concrete made of seawater and/or sea sands will have comparable workability, higher strength in early stages and comparable long-term strength, lower freeze–thaw resistance, almost the same carbonation resistance, more drying shrinkage compared with common concrete. Current research works about FRP-reinforced SWSSC structures are relatively limited. Most works focus on the durability and service life predictions of various FRP products in SWSSC environment or simulated SWSSC environment [16,17,18,19,20,21]. Additionally, there are some published papers concerning the mechanical performance and theoretical models of reinforced SWSSC structures, like SWSSC filled FRP or stainless steel circular columns [22,23,24]. 

The environmental impacts of FRP bar-reinforced SWSSC structures are of great interests. FRP-reinforced SWSSC used in marine structures will not consume fresh water or river sands. And in marine areas, like seacoasts, there will be a shortened transportation distance for sea sands compared with river sands or crushed stones. Also seawater will not be treated specially. In addition, FRP-reinforced SWSSC possesses much longer service lives compared to tradition steel-reinforced structures. This may benefit in reducing environmental impacts. To evaluate the environmental impacts, a life cycle assessment (LCA) study was performed, which is a powerful and internationally accepted method. 

LCA is well defined by ISO 14040 [25] and ISO 14044 [26], and has been widely used in evaluating the environmental impacts of building materials, building technologies and building waste management. Ignacio Zabalza Bribián et al. [27] performed an LCA about building materials and conducted that the environmental impacts of construction products can be significantly reduced through use of the most eco-innovative techniques and products. Pouya Samani et al. [28] did a comparative LCA study between advanced sandwich-structured composites and typical brick house and found that advanced sandwich-structured composites had lower environmental impacts. Gian Andrea Blengini [29] modelled a detailed LCA focusing on the end-of-life phase of building and offering actual data on demolishing and recycling rubble. Results showed building waste recycling was eco-friendly. Besides, LCA methodology has also been used to compare the environmental impacts of different construction systems [30] and external wall systems [31]. Although there is a lot of utilization of LCA in civil engineering, the environment impact assessment of FRP bar-reinforced SWSSC beam used in marine environment has not been studied, which is one of the most promising areas where FRP-reinforced SWSSC structures can be used.

In the present work, three assumed but not real beams (GFRP and CFRP bar-reinforced SWSSC beams and steel rebar-reinforced common concrete beam) were designed under the same specified and reasonable exposed environment as well as loads according to standards including ACI 440.1R-2015 [12], ACI 318-2014 [32], ACI 440.5M-08 [33], ACI 440.6M-08 [34] and ACI 211.1-1991 R2009 [35]. The aim is to illustrate the environmental advantages of FRP bar-reinforced SWSSC beam used in marine environment compared with SRC beam by considering the different design requirements, different durability requirements, different life span and different transportation distance. A cradle-to-grave LCA was used to assess the environmental burdens of the three beams according to standards ISO 14040 and ISO 14044. ReCiPe method and eight environmental categories including climate change (CC), terrestrial acidification (TA), freshwater eutrophication (FREU), fossil depletion (FD), stratospheric ozone depletion (SOD), freshwater eco-toxicity (FREC), human toxicity, cancer (HT) and fine particulate matter formation (FPMF) were used to characterize the environmental impacts of these three beams. Results of the LCA study could support the use of FRP bar-reinforced SWSSC structures in marine environment considering its low environmental impacts.

## 2. Materials and Methods 

### 2.1. Design of Concrete Beams 

To investigate the environmental impacts of FRP bar replacing steel rebar in SWSSC, the load and environment situations were specified previously. The beams are not real but the assumed ones. The specified environment and load situations were used to design the three beams by considering the differences in durability and design criteria like water cement ratio, thickness of concrete cover, deflection limit and so on.

These three beams were designed as non-pre-stressed simply supported beams with rectangular cross sections and one layer of tension reinforcements, which were a GFRP bar-reinforced SWSSC (GFRP-SWSSC) beam, a CFRP bar-reinforced SWSSC (CFRP-SWSSC) beam and a steel rebar-reinforced common concrete (SRC) beam. The span was set as 4 m and the dead load and live load acting on these beams was set as 15 kN/m and 2 kN/m, respectively. The dead load was estimated by assuming the beam beard one quarter of the self-weight of one floor (area of 16 m^2^ and thickness of 100 mm) with leveling materials on it and also the self-weight of a wall (thickness of 200 mm and height of 3 m). The active load was estimated by considering people’s activity. The ratio of the cross-section height to section width of the three beams was set as 2. The exposure category was set as *C*_2_, which means these beams would be exposed to moisture and a source of chlorides [32].

To facilitate the calculation of raw materials consumption for these beams, the maximum size of coarse aggregate *d*_agg_, slump, specific density of cement, bulk specific density and fineness modulus of fine aggregates (sea sands or river sands) were set as 19 mm, 75–100 mm, 3140 kg/m^3^, 2595 kg/m^3^ and 2.8, respectively. In addition, the bulk specific density and dry-rodded density of coarse aggregate were set as 2691 kg/m^3^ and 1602 kg/m^3^, respectively. The density of desalted water and seawater was set as 1000 and 1050 kg/m^3^.

To minimize the effects of design process on the final raw materials consumption of the three beams, there are several design steps to be followed. At first, the cross-section width was estimated by considering the requirements of concrete cover, minimum inside bend diameter of stirrup, minimum straight extension of stirrup and minimum distance between parallel tension reinforcements assuming using 90 degree hook stirrup with the minimum stirrup diameter available. The section height could be calculated then. Finally, check of nominal flexural strength, crack limit, deflection limit, shear strength and creep rupture stress limit were conducted to arrange FRP bars and adjust cross-section sizes for FRP-SWSSC beams. Concerning SRC beam design, check of nominal flexural strength, deflection limit, distribution of steel reinforcement for crack control and shear strength were performed to arrange steel rebar and adjust cross-section size. When half of the factored shear strength provided by concrete exceeds the factored shear strength at the critical section, the maximum allowable spacing was used to place stirrups to hold the tension reinforcements and avoid sudden failure.

Information of sizes and mechanical properties of CFRP and GFRP bars was got from Tables 7.1 and 8.1 in ACI 440.6M-08 [34]. The strength of the CFRP and GFRP stirrup legs were considered to be the same with the strength of straight CFRP and GFRP bars having the same diameters when the ratio of inside bend radius to the diameter of FRP stirrups is greater than 3. 

There are some requirements for both GFRP-SWSSC beam, CFRP-SWSSC beam and SRC beam. Table 1 summarizes these requirements used in the design process. 

### 2.2. Sizes and Amount of Raw Materials of Beams

**Figure 1:**
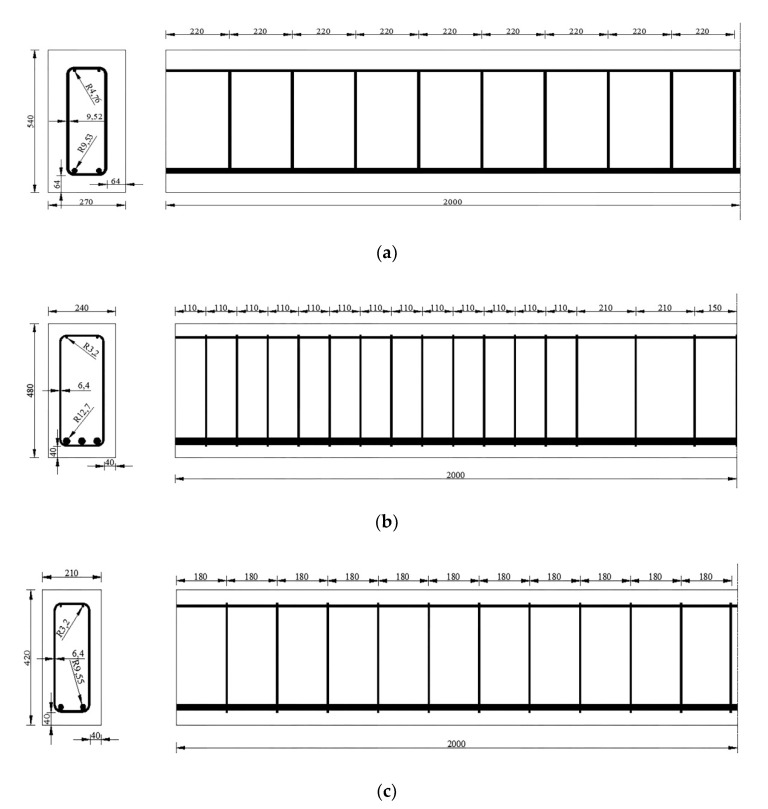
Reinforcements layouts of the half span of (**a**) SRC beam, (**b**) GFRP-SWSSC beam and (**c**) CFRP-SWSSC beam. Unit: mm.

The beams were designed to meet the requirements of both strength and serviceability. Table 2 shows the detailed size parameters and amount of raw materials consumption of the designed beams. Absolute volume basis method was used in computing the mix portion design of both SWSSC and common concrete because according to introduction, concrete made with seawater/sea sands will have a comparative long-term strength. When computing the minimum required average strength, equation from Table 5.2 in ACI 214R-11 was adopted to deal with the problems there are not sufficient historical data. When dealing with the mix portion design for SWSSC, the strict requirements about the minimum specified strength and maximum water cement ratio were not considered. The schemes of these beams are presented by Figure 1.

### 2.3. Life Cycle Assessment

A whole LCA usually includes four steps, i.e., goal and scope definition, life cycle inventory analysis (LCI), life cycle impact assessment (LCIA) and interpretation. In the present study, a professional environmental impacts assessment software GaBi [36] was used to facilitate the whole assessment process.

#### 2.3.1. Goal and Scope Definition

**Figure 2:**
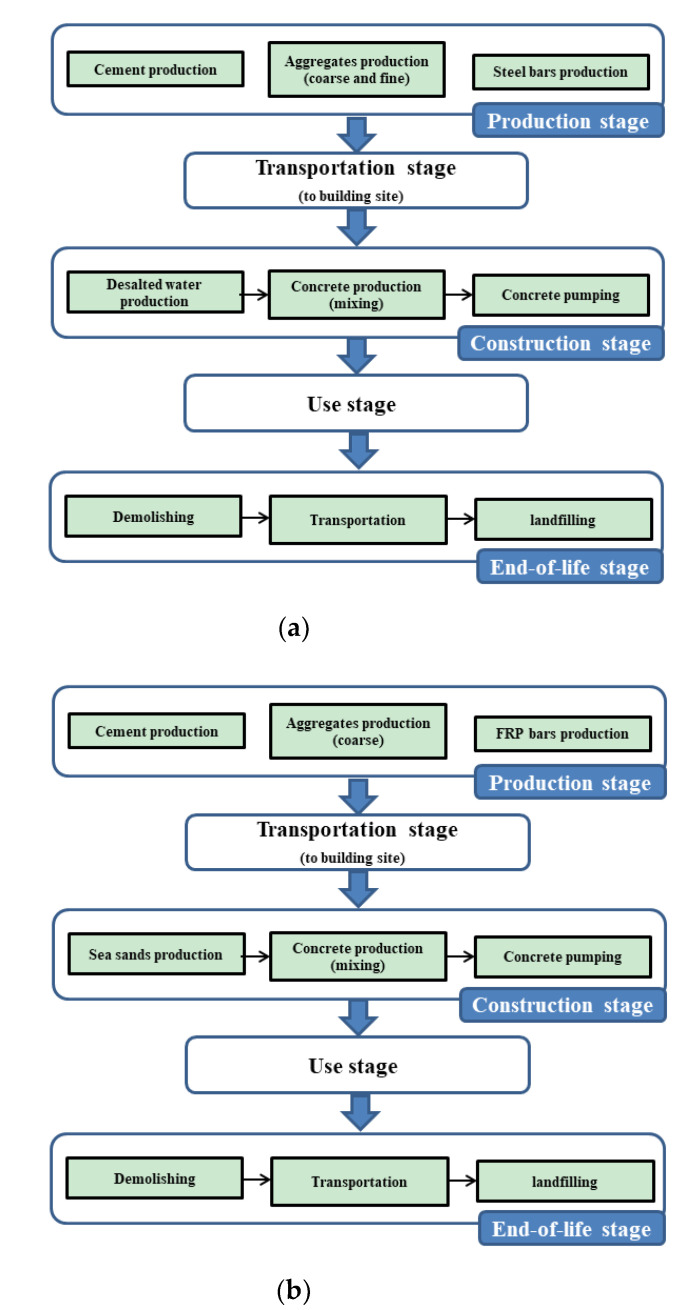
System boundary of (**a**) SRC beam and (**b**) FRP-SWSSC beams.

Goal and scope definition is the first step when performing LCA study. The goal of an LCA study is related to the application of LCA results. The goal of this LCA study is to find the environmental impacts of FRP bar-reinforced SWSSC beams, compare with SRC beam, and to identify the processes which contribute most to the whole environmental impacts of the beams. While scope definition determines which process is included in an LCA study and also specifies the system boundary of the LCA study. The system boundary of the present article is shown in Figure 2.

As for FRP bar-reinforced SWSSC beams, production stage means producing of cement, coarse aggregates, sea sands, FRP tension bars and stirrups. Transportation stage means transporting raw materials to the construction site. Construction stage means mixing and pumping concrete. Use stage means the building is in use and the beam bears load. End-of-life stage means demolishing, transporting waste and landfilling. While for SRC beam, production stage means producing of cement, coarse and fine aggregates, steel bars and desalted water. Other stages are the same with that in FRP bar-reinforced SWSSC beams.

Function unit should also be clearly defined when performing an LCA research because functional unit can connect corresponding inputs and outputs, and offer a basic unit for comparison. The functional unit defined in this study is the beam that meets the requirements of both strength and serviceability, which is similar to that used in [37].

#### 2.3.2. Life Cycle Inventory Analysis (LCI)

Life cycle inventory analysis is the second stage when performing a complete LCA. During this stage, all input and output data related to the whole LCA process should be collected and calculated.

As for SRC beam, the environmental impacts of cement and deionized water production were found in GaBi software. Concerning the production of steel rebar, Table 3 of ref. [38] was used to model its production process in GaBi. According to ref. [39,40], 120 MJ oil and 50 MJ electricity will be consumed when producing one tone of crushed stones. Emissions of oil combustion can be calculated according to Table B.3 in [41]. Table A.8 and B.4 in [41] were used when calculating the environmental impacts of concrete mixing. Concrete pumping process in GaBi software was used to pump the concrete in the construction stage. 

When producing FRP-SWSSC beam, GFRP and CFRP bars should be produced firstly. Epoxy was assumed as the matrix of GFRP and CFRP bars because it is one of the most widely used FRP matrices in civil engineering. The environmental impacts of producing glass fiber, carbon fiber and epoxy resin can be found in GaBi software. Pultrusion process was set as the production method for GFRP and CFRP tension bars with an energy intensity of 3.1 MJ/kg according to [42]. For producing GFRP and CFRP stirrups, filament winding technology was adopted to produce close FRP stirrups, and the energy intensity of filament winding is 2.7 MJ/kg [42]. The fiber volume content of GFRP and CFRP bars was set as 55% according to ACI 440.6M-08. The density of steel rebar, glass fiber, carbon fiber and epoxy is 7.8, 2.5, 1.8 and 1.2 g/cm^3^, respectively, according to [9]. The environmental impacts of cement, coarse aggregates, mixing and demolishing concrete in FRP-SWSSC beams were calculated using the same way with that in the SRC beam.

Since there is no direct environmental impacts data available about the production of sea sands, the environmental impacts of mining sea sand were estimated according to [43,44]. Transportation and extracting processes were considered for the mining of sea sands with a transportation distance of 77.25 km. According to [44], 398.84 kWh electricity would be consumed when 250 cubic meters sands were mined and “electricity from heavy fuel oil” process in GaBi was used to simulate the electricity supply. The bulk density of sea sands was set as 1500 kg/m^3^. For transporting sea sands in GaBi, the “GLO-Bulk commodity carrier-average, ocean going” process was used. The whole sea sands mining process was completed in GaBi software. 

All electricity used in this article was assumed as a mixing power with a power structure of 72.9% thermal power, 18.6% hydropower, 3.9% nuclear power and 4.6% wind power, which is the power structure of China in 2017 [45].

Transportation process was modelled in GaBi software because in GaBi, almost all sorts of transportation processes are covered. The distance between raw material factories to the construction site was set as 50 km at first and then was changed in the sensitivity analysis section to analyze the effects of different transportation distance on the whole environmental impacts of the three beams.

It’s specified that no extra maintenance actions were required in the use phases of these three beams. When computing environmental impacts of the three beams, the life span was set the same at first. Then longer life span of FRP-SWSSC beams will be assumed to investigate the effects of service life on the environmental impacts of these beams.

Considering the end-of-life phase of these three beams, demolition, transportation and landfilling were considered as the final way to handle the construction waste of these beams. Though studies about recycling concrete have been published a lot [46,47,48,49,50,51], according to standard ANEJO 15, the recycled coarse aggregates cannot be incorporated into new concrete when they contain lots of chloride ions, which is the situation in this paper. The energy demanded and emissions to air of demolition were considered referring to [41]. The emissions and diesel consumption for landfilling were got from Table 4 in ref. [50]. The transportation distance between building site and the fixed processing plant where concrete waste can be landfilled was set as 20 km firstly and then it will be altered in the sensitivity analysis section.

#### 2.3.3. Life Cycle Impact Assessment (LCIA)

Life cycle impact assessment phase will connect the results from the second stage with the indicator values of some commonly used environmental impact categories. The environment assessment method used in this work is ReCiPe 2016 v1.1 Midpoint (H) and the environmental categories used include CC, TA, FREU, FD, SOD, FREC, HT and FPMF. 

#### 2.3.4. Interpretation 

Interpretation is the final step when performing an LCA study and in this step, the results from LCIA step should be explained. Conclusions should be drawn according to the scope specified in advance. Recommendations should be proposed to reduce the environmental impacts of the product system studied.

#### 2.3.5. Sensitivity Analysis

In the present study, distance 1 is specified as the distance between raw materials factories and the construction sites while distance 2 is specified as the distance between construction sites and the fixed processing plant where concrete can be recycled or landfilled. As can be seen in ref. [46,50], when the distance 1 and 2 are variable and hard to be determined, sensitivity analysis will be used to find the influences of different distances. By gathering the distance data from ref. [46,47,49,50,51], it is summarized that distance 1 ranges from 24 to 150 km while distance 2 ranges from 5 to 50 km.

## 3. Results and Discussion

### 3.1. Environmental Impacts of SWSSC and Common Concrete of 1 Cubic Meter

**Figure 3:**
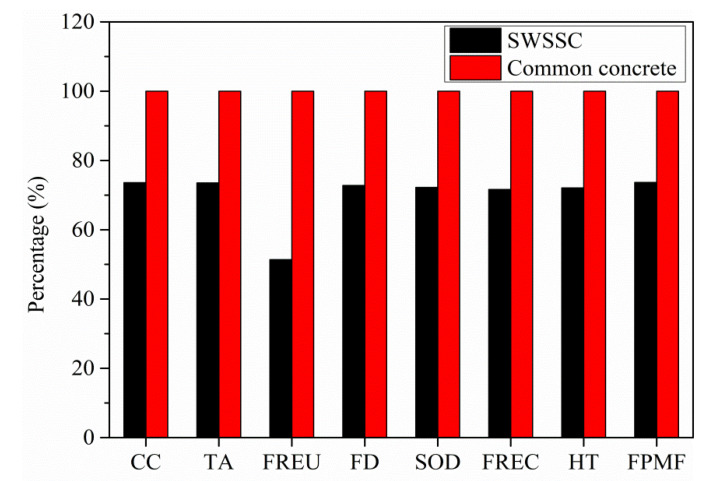
Comparison between environmental impacts of SWSSC and common concrete.

After connecting the outputs of LCI phase with environmental categories, the categories’ indicator values can be calculated. The comparison between environmental impacts of SWSSC and common concrete of 1 cubic meter was shown in Figure 3 without considering transportation. As can be seen from the figure, SWSSC has lower environmental impacts in terms of all the eight categories with a reduction rates from 26.3% to 48.6%. Table 3 presents the categories’ indicator values for the environmental impacts of SWSSC and common concrete of 1 cubic meter.

**Figure 4:**
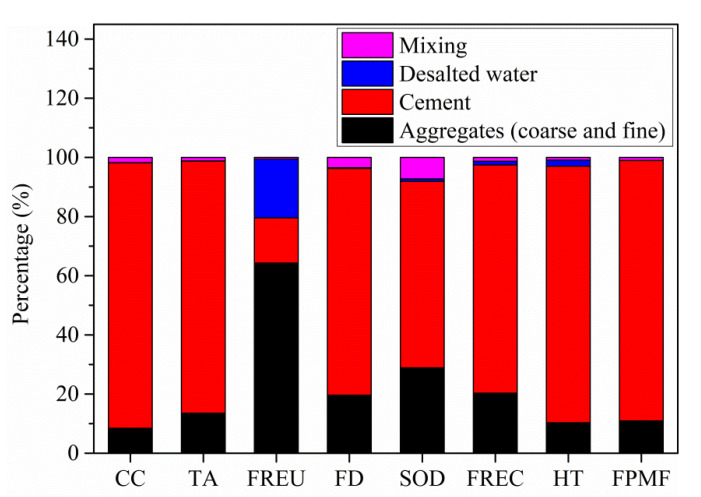
Contribution of different raw materials to the environmental impact category indicator values of common concrete.

Since FRP is corrosion resistant compared with steel rebar, in the present work, the water cement ratio and specified compressive strength of SWSSC were not strictly specified like common concrete. Therefore, there is less cement used in SWSSC, which is the main reason accounting for the difference between environmental impacts of SWSSC and common concrete. The contribution of different raw materials to the environmental impacts of common concrete was shown in Figure 4. As seen, the environmental impacts of cement dominate all the values of environmental impact categories’ indicators of common concrete except category freshwater eutrophication, which is mainly influenced by the environmental impacts of producing aggregates (including both coarse and fine aggregates) and desalted water. The environmental impacts contribution of cement to the eight categories of common concrete is 89.7%, 85.2%, 15.3%, 76.7%, 63.0%, 77.2%, 86.7%, and 88.0%, respectively. The extremely huge effects of cement production on the environmental impacts of common concrete are similar to that in ref. [50].

When considering transportation in marine environment, SWSSC will have less environmental impacts since sea sands do not need to be transported.

### 3.2. Effects of Transportation Distance on the Environmental Impacts of Three Beams

**Figure 5:**
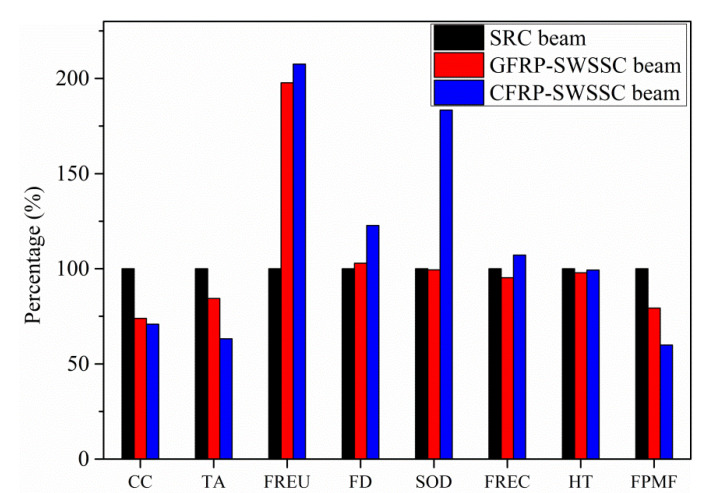
Comparison of environmental impact category indicator values of the three beams with distance 1 and 2 of 50 and 20 km.

When considering the same life span for all the three beams, the environmental impacts of the three beams with distance 1 and distance 2 of 50 km and 20 km, respectively, were shown in Figure 5. Table 4 shows the environmental impact category indicator values of the three beams. As seen, CFRP-SWSSC beam has environmental advantages concerning categories like CC, TA, HT and FPMF with reduction rates of 29.1%, 36.8%, 0.7% and 40.1% compared with SRC beam. GFRP-SWSSC beam possesses advantages concerning categories like CC, TA, SOD, FREC, HT and FPMF with decreasing rates of 26.1%, 15.6%, 0.6%, 4.7%, 2.1% and 20.6% compared with the SRC beam.

When distance 1 and 2 were decreased to 24 and 5 km, assuming FRP-SWSSC beams have the same life span compared with SRC beam, the reduction rates concerning CC, TA and FPMF for CFRP-SWSSC beam are 28.8%, 36.3% and 39.8% compared with SRC beam. For GFRP-SWSSC beam, the reduction rates concerning CC, TA, FREC, HT and FPMF are 25.9%, 15.0%, 3.0%, 1.2% and 20.3%. Table 5 shows the detailed impact category indicator values.

When distance 1 and 2 were increased to 150 and 50 km, assuming FRP-SWSSC beams have the same life span compared with SRC beam, the reduction rates concerning CC, TA, FREC, HT and FPMF for CFRP-SWSSC beam are 30.2%, 38.2%, 1.0%, 4.6% and 41.1% compared with SRC beam. For GFRP-SWSSC beam, the reduction rates concerning CC, TA, FD, SOD, FREC, HT and FPMF are 26.8%, 17.5%, 0.9%, 6.6%, 9.6%, 5.0% and 22.0%. Table 6 shows the detailed impact category indicator values.

From the comparison, CFRP-SWSSC beam and GFRP-SWSSC beam are more likely to reduce environmental burdens when transportation distance increases because they have less weight to be transported compared with SRC beam. The weight of SRC beam, GFRP-SWSSC beam and CFRP-SWSSC beam is 1389.47 kg, 1076.66 kg and 824.19 kg according to Table 2. Therefore, the environmental advantages of FRP-SWSSC beams will become more obvious with the increase of transportation distance.

### 3.3. Effects of Service Life on the Environmental Impacts of the Three Beams

The comparison before was under the assumption that FRP-SWSSC beams have the same life span compared with SRC beam. Actually FRP bar is much more corrosion resistant than steel bar. However, there is not solid statistical data available about the life span of SRC beam and FRP-SWSSC beams under marine environment. The possible environmental impacts reduction of FRP-SWSSC beams can only be investigated based on assumption.

In this study, the effects of different life span on the environmental impacts of the three beams were investigated by assuming FRP-SWSSC beams have 1.5 times longer life span compared with SRC beam, the same way with ref. [52]. Then, the indicator values of eight environmental impact categories for the three beams were shown in Table 7.

As can be seen from the table above, GFRP-SWSSC will have lower category indicator values concerning all the categories except FREU compared with SRC beam. CFRP-SWSSC will have lower category values concerning all the categories except FREU and SOD compared with SRC beam. Therefore, when considering the durability of FRP-SWSSC beams, the environmental advantages of FRP-SWSSC beams will become more obvious.

### 3.4. Environmental Impacts Contribution Analysis

**Figure 6:**
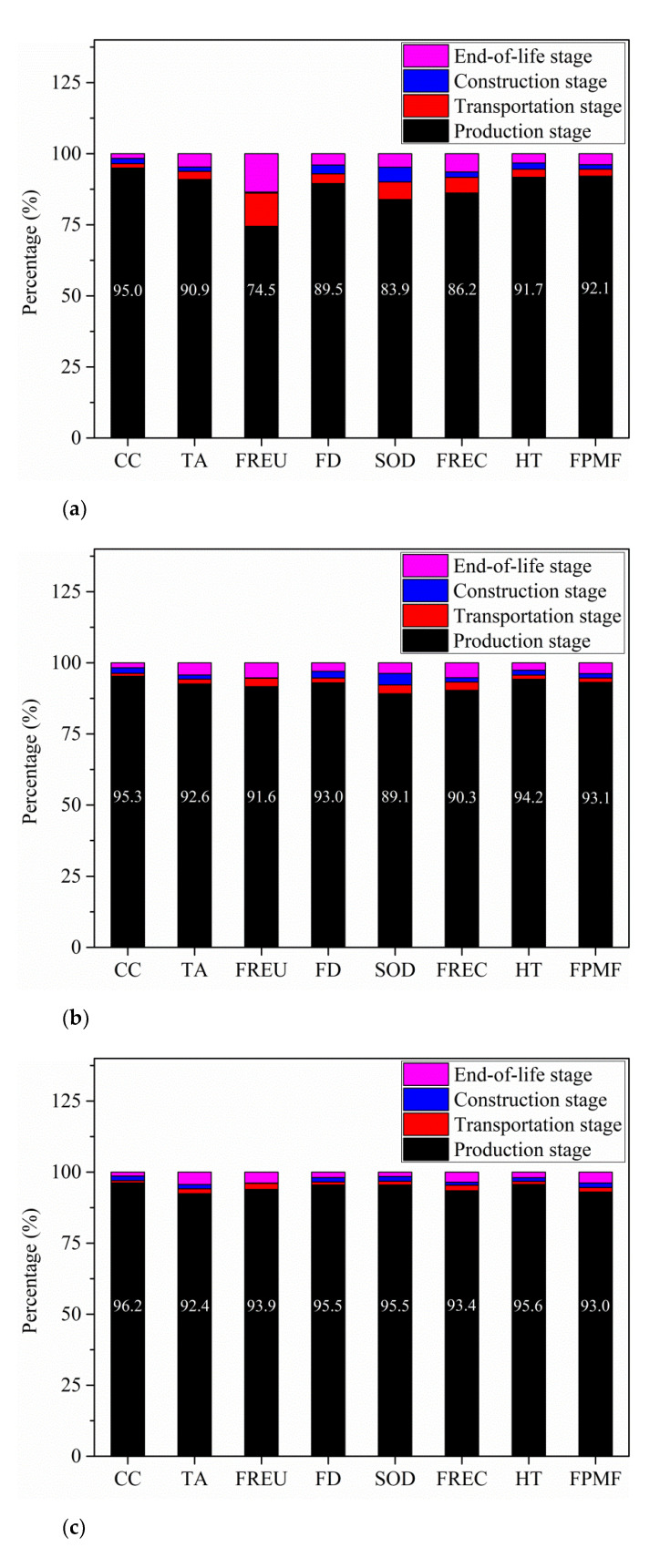
Contribution of different life cycle stages to the whole environmental impacts of (**a**) SRC beam, (**b**) GFRP -SWSSC beam and (**c**) CFRP -SWSSC beam with distance 1 and 2 of 50 and 20 km.

In this part, the environmental impacts contribution of different stages to the total environmental impacts of all the three beams will be analyzed. Figure 6 is the contribution of different stages to the whole life cycle environmental impacts of the three beams.

As can be seen from Figure 6a, the environmental impacts of SRC beam are mainly controlled by the environmental impacts of production stage. Production stage contributes 95.0%, 90.9%, 74.5%, 89.5%, 83.9%, 86.2%, 91.7% and 92.1% to the whole life cycle environmental impacts of SRC beam in terms of CC, TA, FREU, FD, SOD, FREC, HT and FPMF. Transportation stage contributes relatively large to FREU, SOD and FREC with percentage of 11.7%, 6.2% and 5.5%. Contribution of construction stage ranges from 0.4% to 5.2% to the eight categories. Finally, end-of-life stage contributes relatively large to FREU and FREC with percentage of 13.5% and 6.4%.

As can be seen from Figure 6b, the environmental impacts of GFRP-SWSSC beam are mainly affected by the environmental impacts of production stage of this beam too. Production stage contributes 95.3%, 92.6%, 91.6%, 93.0%, 89.1%, 90.3%, 94.2% and 93.1% to the whole life cycle environmental impacts of GFRP-SWSSC beam in terms of CC, TA, FREU, FD, SOD, FREC, HT and FPMF. Contribution of transportation stage, construction stage and end-of-life stage ranges from 1.0% to 3.1%, 0.2% to 4.1% and 1.8% to 5.3%, respectively to the eight categories. Transportation stage contributes less compared with SRC beam, because less weight needs to be transported in GFRP-SWSSC beams.

As seen from Figure 6c, the environmental impacts of CFRP-SWSSC beam are mainly affected by the environmental impacts of production stage of this beam. Production stage contributes 96.2%, 92.4%, 93.9%, 95.5%, 95.5%, 93.4%, 95.6% and 93.0% to the whole life cycle environmental impacts of CFRP-SWSSC beam in terms of CC, TA, FREU, FD, SOD, FREC, HT and FPMF. Contribution of transportation stage, construction stage and end-of-life stage ranges from 0.8% to 2.1%, 0.1% to 1.7% and 1.4% to 4.4% to the eight categories.

## 4. Discussion

There will be more environmental benefits for FRP bar-reinforced SWSSC beams used in marine environment when considering the following factors. At first, FRP bars used in civil engineering has a relatively short development history; therefore, after improving the technology, there will be fewer environmental burdens when producing FRP bars, like the production and use of large tow carbon fiber. Then one of the most important aspects is that use of SWSSC can save river sands or crushed stones resources, which has not been illustrated by the eight categories.

Since FRP is more chloride ions resistant than steel bars, the recycled SWSSC coarse aggregates can be used in new SWSSC again. But for steel-reinforced concrete structures used in marine environment, the concrete cannot be directly recycled into new concrete because of the chloride ions inside it, which may make FRP-reinforced SWSSC structures more eco-friendly.

Life span of the three beams will have vital impacts on the comparison of the environmental impacts between FRP-SWSSC beams and SRC beam. However, the statistical data about the life spans cannot be obtained yet, especially for FRP used in practical projects. Therefore, more research should be done to develop more reasonable life prediction models for FRP-reinforced SWSSC structures in a marine environment.

## 5. Conclusions

In the present paper, the environmental impacts of three beams designed under the same load and environmental situations were evaluated and compared in terms of LCA results. The following conclusions can be drawn based on the results and analysis:

(1) Compared to common concrete in marine environments, SWSSC possesses lower environmental impacts in terms of all eight environmental impact categories with a reduction from 26.3% to 48.6% when considering the differences in design criteria used in marine environments.

(2) If the same life span is assumed with a distance 1 and 2 of 50 and 20 km, compared to SRC beam, the GFRP-SWSSC beam performs better in six categories, and CFRP-SWSSC beam performs better in four categories. When considering the increased transportation distance and improved durability performance, GFRP-SWSSC and CFRP-SWSSC beams shows much more reduced environmental impacts.

(3) The environmental impacts of FRP-SWSSC beams and SRC beam are all mainly affected by the production stages.

## Figures and Tables

**Table 1:** Constructional requirements used in the design process.

Requirements	SRC Beam	FRP-SWSSC Beams
Minimum *C*/mm	63.5 ^1^	40 ^2^
Minimum inside bend diameter of 90-degreee hook stirrups/mm	4 *d*_b_ for bar size from 9.52 to 15.88 ^3^	6 *d*_b_^4^
Minimum straight extension of stirrups/mm	Greater of 6*d*_b_ and 76.2 for bar size from 9.52 to 15.88 ^3^	12 *d*_b_^4^
Minimum distance between tension reinforcements/mm	Greatest of 25.4, *d*_b_, and (4/3) *d*_agg_^5^	Greatest of 25.4, *d*_b_, and (4/3) *d*_agg_^5^
Minimum *f*_c_^’^/MPa	35 ^6^	17.23 ^7^
Maximum water cement ratio	0.4 ^6^	None

^1^ R20.6.1.4.1 in ACI 318-14; ^2^ Table 3.1 in ACI 440.5M-08; ^3^ Table 25.3.2 in ACI 318-14; ^4^ Section 8.3 in ACI 440.1R-15; ^5^ Section 25.2.1 in ACI 318-14; ^6^ Table 19.3.2.1 in ACI 318-14; ^7^ Table 19.2.1.1 in ACI 318-14.

**Table 2:** Size parameters and amount of raw materials of the three beams.

Item	Unit	SRC Beam	GFRP-SWSSC Beam	CFRP-SWSSC Beams
Width of rectangular cross section	mm	270	240	210
Overall height of flexural member	mm	540	480	420
Diameter of tension bar	mm	19.05	25.4	19.1
Diameter of stirrups	mm	9.52	6.4	6.4
*C*	mm	64	40	40
*f*_c_^’^	MPa	35	21	21
Water cement ratio	-	0.4	0.54	0.54
Steel bar	kg	34.30	-	-
Glass fiber	kg	-	10.21	-
Carbon fiber	kg	-	-	3.11
Epoxy resin	kg	-	4.01	1.70
Cement	kg	296.64	172.12	132.74
Desalted water	kg	118.65	-	-
Sea water	kg	-	92.94	71.68
Sea sand	kg	-	347.07	267.67
Aggregates	kg	939.88	450.31	347.29

**Table 3:** Environmental impact category indicator values of 1 m^3^ SWSSC and 1 m^3^ common concrete.

Environmental Impact Category	Unit	SWSSC	Common Concrete
CC	kg CO_2_ eq.	292	397
TA	kg SO_2_ eq.	0.43	0.59
FREU	kg P eq.	6.63 × 10^−5^	1.29 × 10^−4^
FD	kg oil eq.	33.75	46.37
SOD	kg CFC-11 eq.	2.04 × 10^−5^	2.82 × 10^−5^
FREC	kg 1,4 DB eq.	1.21 × 10^−2^	1.69 × 10^−2^
HT	kg 1,4-DB eq.	3.64 × 10^−2^	5.05 × 10^−2^
FPMF	kg PM2.5 eq.	0.16	0.22

**Table 4:** Environmental impact category indicator values of the three beams with distance 1 and 2 of 50 and 20 km.

Environmental Impact Category	Unit	SRC Beam	GFRP-SWSSC Beam	CFRP-SWSSC Beam
CC	kg CO_2_ eq.	256.26	189.39	181.71
TA	kg SO_2_ eq.	0.41	0.35	0.26
FREU	kg P eq.	1.67 × 10^−4^	3.29 × 10^−4^	3.46 × 10^−4^
FD	kg oil eq.	35.47	36.51	43.55
SOD	kg CFC-11 eq.	2.55 × 10^−5^	2.53 × 10^−5^	4.67 × 10^−5^
FREC	kg 1,4 DB eq.	1.25 × 10^−2^	1.20 × 10^−2^	1.35 × 10^−2^
HT	kg 1,4-DB eq.	3.44 × 10^−2^	3.36 × 10^−2^	3.41 × 10^−2^
FPMF	kg PM2.5 eq.	0.15	0.12	9.05 × 10^−2^

**Table 5:** Environmental impact category indicator values of the three beams with distance 1 and 2 of 24 and 5 km.

Environmental Impact Category	Unit	SRC Beam	GFRP-SWSSC Beam	CFRP-SWSSC Beam
CC	kg CO_2_ eq.	252.99	187.40	180.19
TA	kg SO_2_ eq.	0.40	0.34	0.25
FREU	kg P eq.	1.50 × 10^−4^	3.19 × 10^−4^	3.38 × 10^−4^
FD	kg oil eq.	34.44	35.88	43.08
SOD	kg CFC-11 eq.	2.41 × 10^−5^	2.45 × 10^−5^	4.61 × 10^−5^
FREC	kg 1,4 DB eq.	1.20 × 10^−2^	1.16 × 10^−2^	1.32 × 10^−2^
HT	kg 1,4-DB eq.	3.35 × 10^−2^	3.31 × 10^−2^	3.37 × 10^−2^
FPMF	kg PM2.5 eq.	0.15	0.12	8.90 × 10^−2^

**Table 6:** Environmental impact category indicator values of the three beams with distance 1 and 2 of 150 and 50 km.

Environmental Impact Category	Unit	SRC Beam	GFRP-SWSSC Beam	CFRP-SWSSC Beam
CC	kg CO_2_ eq.	266.53	195.23	186.16
TA	kg SO_2_ eq.	0.44	0.36	0.27
FREU	kg P eq.	2.18 × 10^−4^	3.59 × 10^−4^	3.68 × 10^−4^
FD	kg oil eq.	38.69	38.34	44.95
SOD	kg CFC-11 eq.	2.97 × 10^−5^	2.77 × 10^−5^	4.86 × 10^−5^
FREC	kg 1,4 DB eq.	1.44 × 10^−2^	1.30 × 10^−2^	1.43 × 10^−2^
HT	kg 1,4-DB eq.	3.69 × 10^−2^	3.51 × 10^−2^	3.52 × 10^−2^
FPMF	kg PM2.5 eq.	0.16	0.13	9.47 × 10^−2^

**Table 7:** Environmental impact category indicator values of the three beams considering FRP-SWSSC beams having 1.5 times longer service life with distance 1 and 2 of 150 and 50 km.

Environmental Impact Category	Unit	SRC Beam	GFRP-SWSSC Beam	CFRP-SWSSC Beam
CC	kg CO_2_ eq.	399.79	195.23	186.16
TA	kg SO_2_ eq.	0.66	0.36	0.27
FREU	kg P eq.	3.27 × 10^−4^	3.59 × 10^−4^	3.68 × 10^−4^
FD	kg oil eq.	58.04	38.34	44.95
SOD	kg CFC-11 eq.	4.45 × 10^−5^	2.77 × 10^−5^	4.86 × 10^−5^
FREC	kg 1,4 DB eq.	2.16 × 10^−2^	1.30 × 10^−2^	1.43 × 10^−2^
HT	kg 1,4-DB eq.	5.54 × 10^−2^	3.51 × 10^−2^	3.52 × 10^−2^
FPMF	kg PM2.5 eq.	0.24	0.13	9.47 × 10^−2^

## Data Availability

Not applicable.

## Nomenclature

*C*	Clear cover of reinforcement or FRP bars [mm]
*d*_b_	Diameter of reinforcing bar [mm]
*f*_c_^’^	Specified compressive strength [MPa]
